# The Lesions of Syphilis of the Mouth

**Published:** 1899-09

**Authors:** George R. Southwick

**Affiliations:** Boston, Mass.


### THE LESIONS OF SYPHILIS OF THE MOUTH.[1]

BY GEORGE R. SOUTHWICK, M.D., BOSTON, MASS.

It is not the intention of the writer to enter into any detailed description of syphilis of the mouth, but rather to point out the more common manifestations of the disease which suggest precautions against self-infection or the conveyance of the disease to others.

[1] Read before the American Academy of Dental Science, March 1, 1899.

Syphilis is a disease infectious in any of its stages, more so in the primary and secondary forms, less so in the tertiary or hereditary manifestations, but always liable to infect at any time either by direct contact or indirect by some mediate agency. The mucous membrane is so thin and has so many microscopic openings that the syphilitic virus finds ready lodgement, which is rarely the case on the skin unless an abrasion is present. Syphilis may be acquired thus in an innocent manner. Taylor has observed that dentists contract the disease while operating in mouths the seat of mucous patches, and it is fair to add, under such circumstances the virus may be taken from one patient and inoculated on another unless great care is taken to clean and sterilize everything that has touched the patient or has been moistened by the secretions of the mouth. Syphilis is more commonly conveyed to the mouth or face by kissing, bites in quarrels, or, as happened in one case, where a young lady was playfully bitten on her chin by a syphilitic lover.

Sexual perversion, ritualistic circumcision, miscellaneous use of pipes, drinking-cups, razors, towels, eating and glass-blowing utensils, have been responsible for conveying the contagion of syphilis. Mucous membrane is modified skin, and the lesions of it appear about the same time as those of the skin and with a like clinical history, modified by its tonical variations, motion, and moisture. Syphilis is characterized by its reappearance in different forms called stages,—*i.e.*, primary, secondary, and tertiary, all of which are hereditary. These stages, however, are not to be considered as running a definite period of time, but are so irregular as to be rather periods of certain manifestations of the disease. In a general way it may be said that the early forms of syphilis are superficial and disappear without destroying the tissues or leaving tell-tale scars behind them. The lesions which occur late are deep-seated, long-lasting, and are likely to produce extensive ulceration and destruction of tissue with corresponding scars and deformity. White scars or patches in the mouth, about the palate, and on or behind the tonsils should suggest the presence of syphilis, though ulceration may not be present. The primary sore or chancre is less common in the mouth in the secondary lesions. The lip is often the seat of sclerosis, the opposite lip not being affected. The angle of the mouth may be involved, but not so constantly as in the secondary stage. The centre of the ulcer is somewhat depressed, the edges raised, and the base indurated in proportion to the duration of the ulcer. Such a sore disappears without leaving a scar.

Chancres on the tonsil are more common in women than in men. They are accompanied by extensive inflammation and swelling of the adjacent parts, and present the usual local appearance of a chancre. It might be mistaken for a follicular tonsillitis except for the enlargement of neighboring lymphatics, the sharply defined patch with an enormous amount of swelling and induration, and the comparatively healthy condition of the opposite side. The chancre on the skin about the mouth has the usual round form, with indurated base and a swollen hard margin surrounded by an inflamed area; these sores occurring in the hairy parts or where there are many sebaceous glands are apt to be very large and destructive. The cutaneous lesions of the secondary stage are often accompanied and preceded by lesions of the mouth. One of the earliest and most constant manifestations, as well as a very characteristic one, is erythema of the mucous membrane. It seldom produces primarily a lesion, but when it recurs a papule may form. The characteristic type of the erythema of syphilis of the mouth is a livid, diffuse redness of the mucous membrane over the tonsils, sides of the throat, and especially extending as a livid streak down over the arches of the palate. The color may fade from this type into a deeper blush than normal of the mucosa, and be more diffused about the pharynx and palate, where it may bear some resemblance to the pharyngitis of excessive smokers. The papular lesion of the mucous membrane is one of the most characteristic lesions of syphilis, if not the most important. It is known as the mucous patch. It is very infectious and a common source of contagion. It may occur on any exposed mucous membrane. The places of election are the corners of the mouth, the tonsils, and the tip or margin of the tongue. The posterior wall of the pharynx is seldom affected. The typical lesion is a round oval spot, variable in size, with a grayish-white centre slightly raised, and not unlike the color of mucous membrane touched with the nitrate of silver. The border of the ulcer is sharply defined, and the base is infiltrated and indurated. The infiltrated tissue easily breaks down, a characteristic feature of syphilis, which is seen especially in the gummata. This leads to another tell-tale sign of syphilis at the corners of the mouth. The motion of the mouth causes a breaking down of the infiltrated parts of the ulcer, which spreads out in fan-shaped fissures. The result of healing is a scar with radiating lines at the angle of the mouth, almost a sure sign of syphilis. These mucous patches may be seen in the primary stage, and then are superficial and heal without leaving a trace behind them, but are apt to recur in the secondary stage. They are usually single, may be multiple, heal without destruction of tissue, and in themselves are not painful, but cracks or fissures in them in the angle of the mouth or on the tonsils allowing foreign bodies to lodge in them are very painful. The use of tobacco, alcohol, or anything irritating the mucous membrane of the mouth favors the growth of these patches. This is especially seen in the irritation of sharp, irregular teeth against the tongue and cheeks. The base of an ulcer on the tonsil is so uniformly gray as to suggest a diphtheritic deposit, but in diphtheria the patient is manifestly sick, and there is less enlargement of the lymphatics. If in any doubt, take a culture or watch the case for a couple of days. Diphtheria progresses rapidly and the papule slow in comparison. The tongue more than any other part of the mouth shows the lesions of syphilis in all stages. The middle posterior position of the tongue is sometimes affected in the secondary stage by excessive growth of the normal papillæ as were described by Hutchinson as mucous warts, practically condylomata. Smokers and drinkers and those with sharp, irregular teeth are especially apt to suffer with ulceration of the tongue. The papilla becomes prominent in spots, the epithelium exfoliates, and ulcers form, especially on those places subject to irritation; larger areas may be affected with glistening, slightly infiltrated sores, with the formation of superficial cracks. Sometimes round, flat, dense masses form which project a little above the surface, with here and there a papilla elevated and whitish in appearance.

It is well to remember that white scaly patches on the tongue, or buccal mucous membrane, are very apt to mean syphilis. The gums are not so often attacked by papules as other portions of the mouth. They may swell, become infiltrated, and the teeth loosen if the gums ulcerate about them. Gummata are the chief manifestations of tertiary syphilis. They form in soft fibrous or bony tissues. They are nodular masses from the size of a pin to that of a chestnut, ulcerate very easily, and rapidly form deep, ragged, putrid ulcers, which, with large scars, cause much deformity, and in the tongue seriously interfere with its use. The tongue is apt to be the seat of gummata, which give it a lumpy appearance in addition to its hypertrophy. The tongue may also undergo a diffuse hypertrophy without ulceration. The lips, gums, and cheeks are not often the seat of gummata. The lips are the most frequently involved of these, and the gumma is then apt to be seated on the inner surface of the lips, along the attachment of the jaw. The roof of the mouth, particularly at the junction of the hard and soft palate, is a favorite seat for gummata. They ulcerate very rapidly, destroy the periosteum, and necrosis of the bones follows, with perforation into the nasal cavity and extensive loss of substance. This process may be so rapid as to cause perforation in twenty-four hours, and demands prompt recognition and energetic treatment to arrest the destruction of tissue.

Gummata on the posterior wall of the pharynx are rarely primary, and usually secondary to disease in the nares or the isthmus of the pharynx. The ulceration is exceeding rapid and has disastrous results, destroying the palate and replacing it by scar tissue. The large scars seen in the mouth and pharynx are usually the result of the healing of ulcerated gummata. The lesions of syphilis of the nose and larynx are similar to those of the mouth, with the addition that in the nose a chronic coryza with a very fluid discharge is a suggestion of syphilis, particularly in children, and the ulceration of the gumma produces an exceedingly offensive odor. Syphilis of the larynx interferes with the normal action of the larynx, and the voice has a metallic sound which is diagnostic to the ear of a trained observer. The laryngoscope readily shows the mucous patches or an ulcerating gumma, which is most frequently on the epiglottis or sides of the larynx. The secretions in the mouth are infectious in either nasal or laryngeal syphilis, when mucous patches or ulcers are present. Hereditary syphilis about the mouth most often attacks the bony structure. The teeth are commonly affected, and great importance was attached to the condition of the teeth by Hutchinson, who described the condition which has been termed the Hutchinson teeth. The first teeth are lost early, either from inflammation of the tooth-sacs and exfoliation of the crowns, or, as Hutchinson says, the neck of the tooth has been destroyed by caries and the crown breaks off. The permanent central incisors are regarded as the test-teeth for inherited syphilis. The type of such an inheritance is characterized by narrowing of the cutting edge, the tooth having a peg-like shape with a single crescent-like notch or upward curve across the cutting edge of the tooth. The teeth are rounded, dwarfed, and have an interspace so they do not touch. These teeth are characteristic of inherited syphilis, but precisely the same conditions are found sometimes in the non-syphilitic, and the sign is of positive value only when other confirming signs of syphilis are present. Caries of the alveolar processes, or of the body of the superior maxillary in children, is very apt to be due to hereditary syphilis. It sometimes simulates closely disease in the antrum. Such cases may show a whitish cloud in the cornea, the result of inflammation, or an irregular pupil which does not dilate promptly or evenly, the sequence of a syphilitic iritis. Not every sore on the mouth or in it is syphilis, but every one with a grayish indurated base and a sharply defined border will furnish a good reason for personal precaution, such as wearing rubber cots on the fingers, and the careful sterilization of instruments. Cold sores and herpes labialis are distinguished by their vesicular appearance at first, followed by scabbing, and are situated on the cutaneous rather than on the mucous surface. Epithelial cancer may bear so close a resemblance to a syphilitic ulcer that the unaided eye cannot distinguish the difference. The pain is greater, and enlargement of the glands at the angle of the jaw is more constant and more pronounced in syphilis. The epithelioma is more common between forty and fifty years of age, and in smokers. It usually has a dry, brown or black scab on the surface, covering in the beginning a warty-looking spot, or later an irregular ulcer with sharply defined borders and a red base. Specific treatment for syphilis will soon clear up the diagnosis, or, if haste is urgent, the microscope will decide the question.

It is noteworthy that leucoplacia of the tongue and the scars following the healing of gummata are often the seat of cancer in later life. I have seen one case of ulceration on the alveolar process, just back of the last molar in the lower jaw, which showed some resemblance to syphilis and had apparently some connection with a very large amalgam filling. The ulcer was two-thirds the size of a penny, with a white, excavated base. The gentleman was an excessive smoker and had been treated ineffectually by some of the best specialists in Boston. He said the original filling had been removed and replaced by gutta-percha. He improved under my care, but was not cured. Specific treatment did not benefit him, though the ulcer bore a little resemblance to some of the forms of syphilis of the mouth I have seen. Cancer was excluded by the history of the case. He had some trouble with the soft filling, and called on another dentist, who found the original filling had been capped only with gutta-percha. He removed all the amalgam from an exceedingly large cavity and filled again with gutta-percha. The ulcer healed in about ten days and my patient had no more trouble.

